# Attenuation of Free Fatty Acid-Induced Muscle Insulin Resistance by Rosemary Extract

**DOI:** 10.3390/nu10111623

**Published:** 2018-11-02

**Authors:** Filip Vlavcheski, Evangelia Tsiani

**Affiliations:** 1 Department of Health Sciences, Brock University, St. Catharines, ON L2S 3A1, Canada; fvlavcheski@brocku.ca; 2 Centre for Bone and Muscle Health, Brock University, St Catharines, ON L2S 3A1, Canada

**Keywords:** muscle, insulin resistance, free fatty acids (FFA), diabetes, rosemary extract, AMPK

## Abstract

Elevated blood free fatty acids (FFAs), as seen in obesity, impair muscle insulin action leading to insulin resistance and Type 2 diabetes mellitus. Serine phosphorylation of the insulin receptor substrate (IRS) is linked to insulin resistance and a number of serine/threonine kinases including JNK, mTOR and p70 S6K have been implicated in this process. Activation of the energy sensor AMP-activated protein kinase (AMPK) increases muscle glucose uptake, and in recent years AMPK has been viewed as an important target to counteract insulin resistance. We reported recently that rosemary extract (RE) increased muscle cell glucose uptake and activated AMPK. However, the effect of RE on FFA-induced muscle insulin resistance has never been examined. In the current study, we investigated the effect of RE in palmitate-induced insulin resistant L6 myotubes. Exposure of myotubes to palmitate reduced the insulin-stimulated glucose uptake, increased serine phosphorylation of IRS-1, and decreased the insulin-stimulated phosphorylation of Akt. Importantly, exposure to RE abolished these effects and the insulin-stimulated glucose uptake was restored. Treatment with palmitate increased the phosphorylation/activation of JNK, mTOR and p70 S6K whereas RE completely abolished these effects. RE increased the phosphorylation of AMPK even in the presence of palmitate. Our data indicate that rosemary extract has the potential to counteract the palmitate-induced muscle cell insulin resistance and further studies are required to explore its antidiabetic properties.

## 1. Introduction

Insulin plays a critical role in maintaining blood glucose homeostasis. The increase in postprandial glucose levels causes the release of insulin by the β cells of the pancreas which is delivered to its target tissues via the bloodstream. In skeletal muscle and adipose tissue, insulin promotes the transport, utilization and storage of glucose [1,2], while in the liver, insulin inhibits endogenous glucose production. The end result of these actions of insulin is to return the plasma glucose levels to a physiological range of 4–7 millimolar (mM).

The action of insulin in muscle cells is initiated by binding to its receptor, leading to tyrosine phosphorylation of the receptor and insulin receptor substrate (IRS-1), activation of the lipid kinase phosphatidylinositol-3 kinase (PI3K) and the serine threonine kinase Akt resulting in GLUT4 glucose transporter translocation from an intracellular pool to the plasma membrane and increase in glucose uptake [3,4]. Impairments in the PI3K-Akt cascade leads to insulin resistance and type 2 diabetes mellitus (T2DM) [1,2,5].

Skeletal muscle accounts for around 80% of postprandial glucose uptake and is quantitatively the most important insulin target tissue, and therefore muscle insulin resistance is a major contributor to decreased glucose tolerance and T2DM. Insulin resistance is strongly associated with obesity and increased plasma lipid levels. In vitro studies have shown that exposure of muscle cells to the free fatty acids (FFA) palmitate induces insulin resistance [6]. In addition, evidence from in vivo animals studies have shown that lipid infusion [7,8] or increased plasma lipid levels by high fat diet results in muscle insulin resistance [7,9]. Studies have shown that serine phosphorylation of IRS-1 leads to impairment in the insulin-signaling pathway and contributes to insulin resistance [6,10,11]. Signaling molecules such as mammalian target of rapamycin (mTOR) [12,13], ribosomal protein S6 kinase (p70 S6K) [14,15], glycogen synthase kinase 3 (GSK3) [16], c-Jun N-terminal kinase (JNK) [17] and protein kinase C (PKCs) [18] have been implicated in the serine phosphorylation of IRS-1 [19].

Adenosine monophosphate (AMP)-activated protein kinase (AMPK) is a serine/threonine kinase acting as a cellular energy sensor and activated by increased AMP/ATP ratio and/or via phosphorylation by its upstream kinases, liver kinase B1 (LKB1), calmodulin-dependent protein kinase (CaMKKs) and transforming growth factor-β (TGF-β)-activated kinase 1 (TAK1) [20,21]. Muscle AMPK is activated by muscle contraction/exercise [21] and several compounds including metformin [22], thiazolidineones [23] and polyphenols such as resveratrol [24] and naringenin [25] leading to increased glucose uptake. In recent years, AMPK activators have been recognized as promising pharmacological intervention for the prevention and treatment of T2DM [21,26,27,28].

Rosemary (*Rosmarinus officinalis* L.) is an aromatic evergreen plant reported to have antioxidant [29,30], anticancer [19,20] and antidiabetic properties [31,32,33,34,35,36]. Rosemary extract (RE) contains different classes of polyphenols including phenolic acids, flavonoids and phenolic terpenes [37]. The polyphenols found in the highest quantity in RE are carnosic acid (CA), carnosol (COH) and rosmarinic acid (RA) and their production is influenced by growth conditions such as soil quality, water availability and sunlight exposure. Furthermore, the choice of solvent and extraction method affects the chemical composition of the extract with the possibility of losing lipid soluble chemicals by an aqueous-based extraction method and water-soluble chemicals by non-polar solvent (ethanol, methanol)-based extraction.

Previous studies by our group found a significant increase in muscle glucose uptake and AMPK activation by RE treatment [38]. In addition, administration of RE decreased plasma glucose levels in streptozotocin-induced diabetic mice [31], rats [33,35,36], alloxan-induced diabetic rabbits [32], genetic [34], and dietary [36,39,40,41] animal models of obesity and insulin resistance.

According to the World Health Organization and the International Diabetes Federation (IDF) estimates, T2DM is a disease on the rise [42] and with huge economic burden to health care systems around the globe. Although many different strategies currently exist for the prevention and treatment of insulin resistance and T2DM, they are lacking in efficacy and, therefore, there is a need for new preventative measures and targeted therapies. In recent years, chemicals found in plants/herbs have attracted attention for their use as functional foods or nutraceuticals for preventing and treating insulin resistance and T2DM.

In the present study, we focused on RE and examined its potential to counteract the palmitate-induced insulin resistance in muscle cells.

## 2. Materials and Methods

### 2.1. Materials

Fetal bovine serum (FBS), dimethyl sulfoxide (DMSO), palmitate, bovine serum albumin (BSA) and cytochalasin B, were purchased from Sigma Life Sciences (St. Louis, MO, USA). Materials for cell culture and trypan blue solution 0.4% were purchased from GIBCO Life Technologies (Burlington, ON, USA). Phospho—and total AMPK (CAT 2531 and 2532, respectively), Akt (CAT 9271 and 9272 respectively), JNK (CAT 9251 and 9252, respectively), mTOR (CAT 2971 and 2972, respectively), p70 S6K (CAT 9205 and 2708, respectively) and HRP-conjugated anti-rabbit antibodies (CAT 7074) were purchased from New England BioLabs (NEB) (Missisauga, ON, Canada). Insulin (Humulin R) was from Eli Lilly (Indianapolis, IN, USA). Luminol Enhancer reagents, polyvinylidene difluoride (PVDF) membrane, reagents for electrophoresis and Bradford protein assay reagent were purchased from BioRad (Hercules, CA, USA). [3H]-2-deoxy-d-glucose was purchased from PerkinElmer (Boston, MA, USA).

### 2.2. Preparation of Rosemary Extract (RE)

Following previously established protocols by our group [38] whole dried rosemary leaves (*Rosmarinus officinalis* L.) (Compliments, Sobey’s Missisauga, ON, Canada) were grounded and passed through a mesh sieve. 5 grams of ground leaves were steeped for 16 h in dichloromethane-methanol (1:1) (30 mL). Under a slight vacuum the filtrate was collected followed by methanol (30 mL) extraction for 30 min. The solvent was removed using rotary evaporator. Aliquots of the extract dissolved in dimethyl sulfoxide (DMSO) were prepared (100 μg/mL) and were stored at −20 °C. All experiments were performed using the same batch of RE.

### 2.3. Preparation of Palmitate Stock Solution

Stock palmitate solution was prepared by conjugating palmitate with fatty acid-free BSA as previously reported [6]. In brief palmitic acid was dissolved in 0.1N NaOH and diluted in 9.7% *(w*/*v)* BSA solution that was previously warmed (45–50 °C) to give a stock solution of 8 mM palmitate. The final molar ratio of free palmitate/BSA was 6:1.

### 2.4. Cell Culture, Treatment and Glucose Uptake

L6 rat muscle cells were used in all experiments. Myoblasts were grown and differentiated into myotubes, as previously established [5,24]. Briefly, cells were grown in α-Minimum Essential Medium (MEM) media containing 2% *v*/*v* FBS until fully differentiated. Myotube stage was reached at approximately 6 to 7 days after seeding. All treatments were performed using serum-free media. The fully differentiated myotubes were treated with 0.2 mM palmitate in the absence or presence of 5 μg/mL RE for 16 h followed by treatment without or with 100 nM insulin for 0.5 h. A vehicle-treated control DMSO group was used in parallel with the treated groups. Following the treatment, the cells were rinsed using HEPES-buffered saline (HBS) and exposed to HBS containing 10 μM [3*H*]-2-deoxy-d-glucose for 10 min to measure glucose uptake, as previously described [24,43]. Cytochalasin B (10 μM) was used to determine the non-specific glucose uptake. Cells were seeded in 12-well plates and 3 wells were used for each treatment group. The first two wells were used to measure the total glucose uptake and the third used to measure the non-specific (treated with cytochalasin B). The two total glucose uptake values were averaged, and the non-specific value was subtracted to obtain the specific. At the end of the assay, the cells were rinsed with 0.9% NaCl solution and lysed using 0.05 N NaOH. The radioactivity was measured by liquid scintillation counter and the Bradford assay was used to examine the cellular protein content.

### 2.5. Immunoblotting

After treatment, the cells were quickly washed with ice cold HBS solution and lysed using cold lysis buffer. Whole cell lysates were prepared using lysis buffer containing 20 mM Tris (PH 7.5), 150 mM NaCI, 1 mM ethylenediaminetetraacetic acid (EDTA), 1 mM ethylene glycol-bis β-aminoethyl ether/egtazic acid (EGTA), 1% Triton X-100, 2.5 mM sodium pyrophosphate, 1 mM p-glycerolphosphate, 1mM sodium orthovanadate (Na_3_VO_4_), 1 µg/mL leupeptin. Phenylmethylsulfonyl fluoride (PMSF) was added, to a final concentration of 1 mM, prior to use. The lysates were stored at −20 °C. The protein samples (20 µg) were separated using sodium dodecyl sulfate polyacrylamide gel electrophoresis (SDS-PAGE) followed by a transfer to a PVDF membrane. The membranes were blocked using blocking buffer (5% (*w*/*v*) dry milk powder in Tris-buffered saline) followed by overnight incubation at 4 °C with the primary antibody. The primary antibody was detected using HRP-conjugated anti-rabbit secondary antibody followed by exposure to LumiGLOW reagent. The corresponding bands were visualized with FluroChem software (Thermo Fisher, Waltham, MA, USA).

### 2.6. Statistical Analysis

Statistical analysis was completed using GraphPad Prism software 5.3 manufactured from Graphpad Software Inc. (La Jolla, CA, USA). The data from several experiments were pooled and presented as mean ± standard error (SE). The means of all the groups were obtained and compared to the control group using one-way analysis of variance (ANOVA) which was followed by Tukey’s post hoc test for multiple comparisons.

## 3. Results

### 3.1. Rosemary Extract Restores the Insulin-Stimulated Glucose Uptake in Palmitate-Treated Muscle Cells

**Figure 1:**
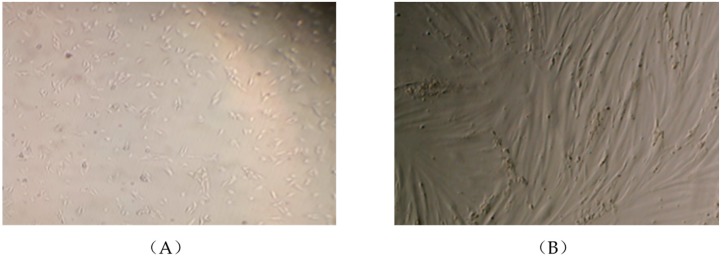
L6 muscle cells in the myoblast (**A**) and fully differentiated myotube (**B**) stage. L6 cells were seeded and cultured in 2% fetal bovine serum (FBS)-containing α-MEM culture media (day 1, **A**) and upon reaching confluency were spontaneously differentiated into myotubes (day 7, **B**). Photographs were taken using EVOS XL Core imaging system at magnification ×10 and ×20.

All the experiments were performed using L6 cells in their differentiated myotube stage (Figure 1). In our lab we have used L6 myotubes for different studies extensively for more than 20 years and differentiation of the cells is assessed microscopically. We are certain that all experiments were performed using differentiated cells/myotubes. Upon differentiation of L6 cells, the expression of the insulin receptor and GLUT4 transporters dramatically increases which results in a 2-fold increase in insulin responsiveness. We examined routinely the response of the cells to insulin (100 nM for 30 min) and we got a 2-fold increase in glucose uptake an indirect measurement of differentiation. Altogether with (1) the microscopic evaluation and (2) the biological evaluation/ insulin responsiveness/glucose uptake assay we are absolutely sure that the cells used in the present study were at the myotube stage.

**Figure 2:**
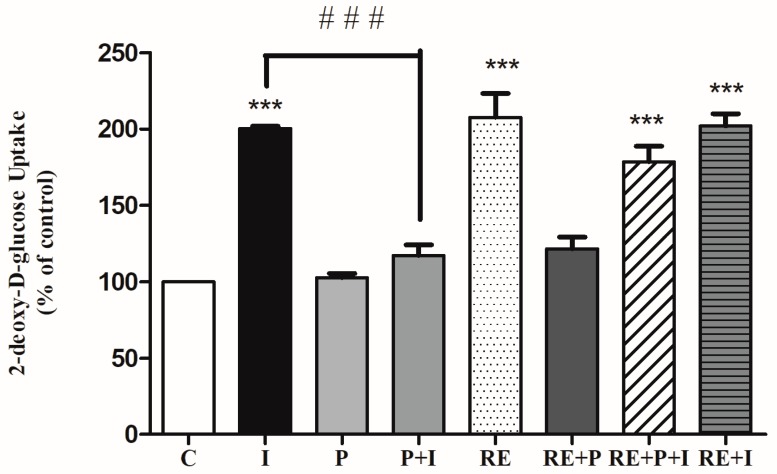
Rosemary extract (RE) restores the insulin-stimulated glucose uptake in palmitate treated muscle cells. Fully differentiated L6 myotubes were treated without (control, C) or with 0.2 mM palmitate (P) for 16 h in the absence or the presence of 5 μg/mL RE followed by stimulation without or with 100 nM insulin (I) for 30 min and [3*H*]-2-deoxy-d-glucose uptake measurements. The results are the mean ± standard error (SE) of 4–7 independent experiments, expressed as percent of control (*** *p* < 0.001 vs. control, ### *p* < 0.001 vs. insulin alone).

The effects of the free-fatty acid palmitate in the absence or the presence of RE on the insulin-stimulated glucose uptake was examined. Acute stimulation of L6 myotubes with insulin (100 nM, 30 min) significantly increased glucose uptake (201 ± 1.21% of control, *p* < 0.0001, Figure 2). Exposure of the cells to palmitate (0.2 mM, 16 h) although did not have any effect on the basal glucose uptake (103 ± 2.7% of control) (Figure 2), it resulted in significant reduction of the insulin-stimulated glucose uptake (117 ± 15.6% of control) indicating insulin resistance. Most importantly in palmitate-treated cells, exposure to RE resulted in significant restoration of insulin-stimulated glucose uptake (179 ± 10.5% of control, *p* = 0.0001, Figure 2). Exposure of the cells to RE (5 µg/mL) alone resulted in significant increase in glucose uptake (208 ± 15.6% of control, *p* < 0.0001). Treatment with RE and palmitate did not have a significant effect on glucose uptake (122 ± 7.6% of control). Moreover, combined treatment with RE and insulin (202 ± 8.0% of control, *p* < 0.0001) did not result in greater response than each treatment alone. These data indicate that the negative effect imposed by palmitate treatment on insulin responsiveness is abolished by the presence of RE.

To investigate any potential cell-damaging effects of palmitate and RE treatment, we examined cell morphology and cell viability. No changes in cell morphology was seen with any of the treatments. Additionally, we utilized the trypan blue exclusion assay to examine cell viability. No effect on cell viability (RE: 98%, P: 97%, P + RE: 99% of control) was seen.

### 3.2 Rosemary Extract Prevents the Palmitate-Induced Ser307 and Ser636/639 Phosphorylation of IRS-1

**Figure 3:**
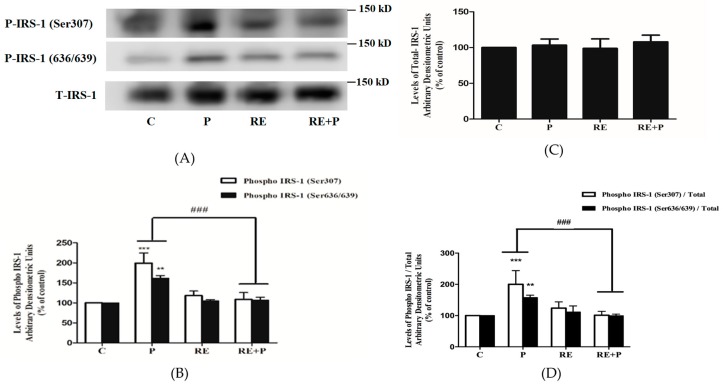
Effects of palmitate and RE on IRS-l expression and Ser307, Ser636/639 phosphorylation. Fully differentiated myotubes were treated without (control, C) or with 0.2 mM palmitate (P) in the absence or the presence of 5 μg/mL RE for 16 h. After treatment, the cells were lysed, and sodium dodecyl sulfate polyacrylamide gel electrophoresis (SDS-PAGE) was performed, followed by immunoblotting with specific antibodies that recognize phosphorylated (Ser307, Ser636/639) or total IRS-1 (T-IRS-1). Representative immunoblots are shown (**A**). The densitometry of the bands was measured and expressed in arbitrary units (**B**–**D**). The data are the mean ± SE of three separate experiments (*** *p* < 0.001, ** *p* < 0.01 vs. control, ### *p* < 0.001 vs. palmitate alone).

Previous studies conducted in L6 muscle cells in vitro and rat muscle in vivo have indicated that increased phosphorylation levels of Ser307 and Ser636/639 of IRS-1 leads to impairment in the insulin signaling leading to insulin resistance [44,45]. Therefore, next we investigated the effects of palmitate and RE downstream of the insulin receptor and examined IRS-1 phosphorylation and expression. Exposure of L6 myotubes to 0.2 mM palmitate resulted in significant increase in Ser307 and Ser636/639 phosphorylation of IRS-1 (199.4 ± 24.98%, 162 ± 6.74% of control, *p* = 0.0005, *p* < 0.0091 respectively) (Figure 3A,B). Treatment with 5 µg/mL RE did not have any effect on the basal Ser307 or Ser636/639 phosphorylation (118 ± 11.24%, 105 ± 3.51% of control respectively) but completely abolished the palmitate-induced increase in Ser307 and Ser636/639 phosphorylation of IRS-1 (108 ± 16.91% of control and 107 ± 7.32% of control, respectively), (Figure 3A,B). The total levels of IRS-1 were not impacted by any treatment (P: 103.3 ± 8.63, RE: 98.82 ± 13.21, RE + P: 108 ± 9.33) (Figure 3C). The ratio of phosphorylated levels of IRS-1 (Ser307 and Ser636/639) over the total levels of IRS-1 is shown on Figure 3D. Treatment with palmitate significantly increased the ratio of phosphorylated Ser307 and Ser636/639 /total IRS-1(202.2 ± 43.7, 157.7 ± 7.7% of control, *p* < 0.0009 respectively). RE did not have an effect on the ratio of basal Ser307 and Ser636/639 phosphorylation of IRS-1/ total IRS-1 (123.8 ± 20 and 111.4 ± 19.4% of control respectively) but completely abolished the palmitate-induced response (101.1 ± 12.6 and 99.3 ± 5.1% of control, *p* = 0.0008 and *p* = 0.0086 respectively).

### 3.3. Rosemary Extract Restores the Insulin-Stimulated Akt Phosphorylation in Palmitate Treated Myotubes

**Figure 4:**
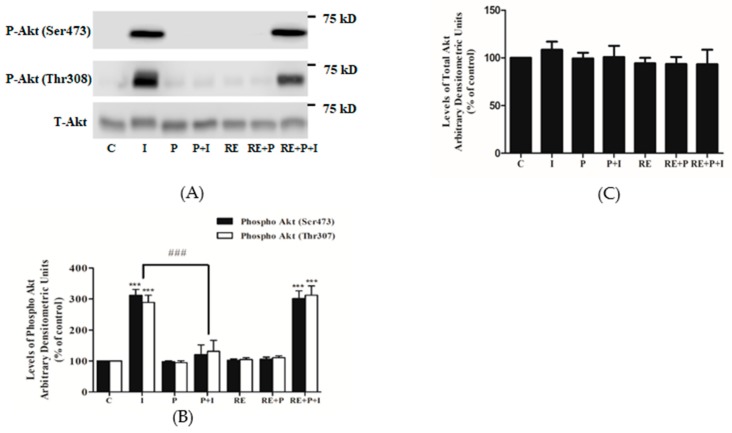
Effects of palmitate and RE on Akt expression and Ser473 and Thr308 phosphorylation. Fully differentiated L6 myotubes were treated without (control, C) or with 0.2 mM palmitate (P) for 16 h in the absence or the presence of 5 μg/mL RE followed by stimulation without or with 100 nM insulin (I) for 15 min. After treatment, the cells were lysed, and SDS-PAGE was performed, followed by immunoblotting with specific antibodies that recognize phosphorylated Ser473, Thr308 or total Akt. Representative immunoblots are shown (**A**). The densitometry of the bands was measured and expressed in arbitrary units (**B**,**C**). The data are the mean ± SE of three separate experiments (*** *p* < 0.001 vs. control, ### *p* < 0.001 vs. insulin alone).

Next, we investigated the effect of palmitate and RE treatment on insulin-stimulated Akt phosphorylation and expression. Treatment of L6 myotubes with insulin resulted in a significant increase in Akt Ser473 and Thr308 phosphorylation (I: 312 ± 19.21 and 289 ± 23.12% of control, *p* = 0.0006, *p* = 0.0009, respectively) (Figure 4A,B). Treatment of the cells with palmitate abolished the insulin-stimulated Akt phosphorylation on Ser473 and Thr308 residues (P + I: 121.9 ± 31.30 and 131 ± 35.90% of control respectively, *p* = 0.0008) (Figure 4A,B). Palmitate and RE each alone or in combination did not have any effect on the basal Ser473 or Thr308 Akt phosphorylation (P: 98.2 ± 3.02, 95 ± 6.20, RE: 103 ± 4.10, 105 ± 6.2%, RE + P: 109.1 ± 9.06, 111 ± 5.92% of control, respectively). However, in the presence of RE, the decline in the insulin-stimulated Akt phosphorylation on Ser473 and Thr308 seen with palmitate was completely prevented (RE + P + I: 346.7 ± 66 and 312 ± 30.31% of control respectively, *p* < 0.001 (Figure 4A,B). The total levels of Akt were not significantly affected by any of the treatments (I: 108 ± 8.4, P: 99 ± 5.9, P + I: 101 ± 11.6, RE: 94 ± 5.72, RE + P: 93.6 ± 7.2, RE + P + I: 93 ± 15.23% of control) (Figure 4C).

### 3.4. Rosemary Extract Prevents the Palmitate-Induced Phosphorylation of C-Jun N-Terminal Kinase (JNK) in L6 Myotubes

**Figure 5:**
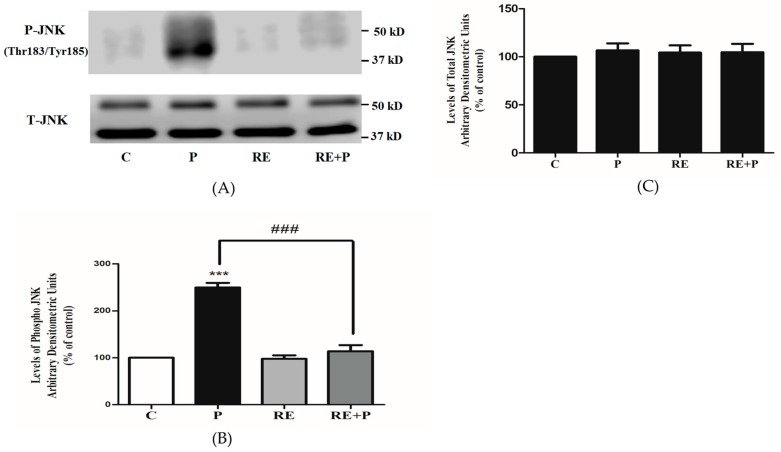
Effects of palmitate and RE on JNK expression and phosphorylation. Fully differentiated myotubes were treated without (control, C) or with 0.2 mM palmitate (P) for 16 h in the absence or the presence of 5 μg/mL RE. After treatment, the cells were lysed, and SDS-PAGE was performed, followed by immunoblotting with specific antibodies that recognize phosphorylated Thr183/Tyr185 or total JNK. Representative immunoblots are shown (**A**). The densitometry of the bands was measured and expressed in arbitrary units (**B**,**C**). The data are the mean ± SE of three separate experiments (*** *p* < 0.001 vs. control, ### *p* < 0.001 vs. palmitate alone).

Following the establishment that chronic exposure to palmitate increases the phosphorylation of Ser307 and Ser636/639 of IRS-1, we examined the signaling molecules that may be involved. JNK is a serine/threonine kinase shown to increase serine phosphorylation of IRS-1 and involved in insulin resistance [46,47]. We hypothesized that the levels of JNK phosphorylation and/or expression would be increased by palmitate. Indeed, exposure of the cells to palmitate (0.2 mM) significantly increased JNK phosphorylation (250 ± 9.77% of control, *p* = 0.0007) and treatment with RE completely abolished the palmitate-induced phosphorylation of JNK (114 ± 12.90% of control, *p* = 0.0006) (Figure 5A,B). RE alone did not affect the phosphorylation of JNK (98 ± 7.44% of control). Moreover, the total levels of JNK were not significantly changed by any treatment: P: 107 ± 7.21, RE: 104 ± 7.53 and RE + P: 105 ± 8.76% of control (Figure 5C).

### 3.5. Rosemary Extract Prevents the Palmitate-Induced Phosphorylation of mTOR and p70 S6K in L6 Myotubes

**Figure 6:**
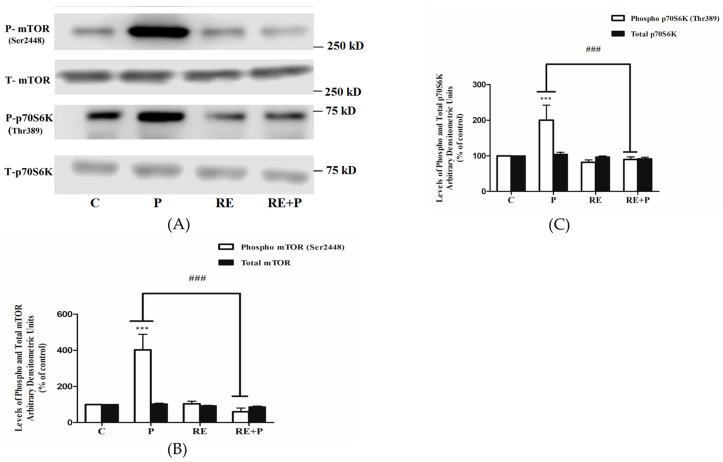
Effects of palmitate and RE on mTOR and p70 S6K expression and phosphorylation. Fully differentiated myotubes were treated without (control, C) or with 0.2 mM palmitate (P) for 16 h in the absence or the presence of 5 µg/mL RE. After treatment, the cells were lysed, and SDS-PAGE was performed, followed by immunoblotting with specific antibodies that recognize phosphorylated Ser2448 or total mTOR or phosphorylated Thr389 or total p70 S6K. Representative immunoblots are shown (**A**). The densitometry of the bands was measured and expressed in arbitrary units (**B**,**C**). The data are the mean ± SE of three separate experiments (*** *p* < 0.001 vs. control, ### *p* < 0.001 vs. palmitate alone).

Another kinase implicated in serine phosphorylation of IRS-1 is mTOR and, therefore, we examined the effects of palmitate on mTOR phosphorylation/activation and expression. Exposure of the cells to 0.2 mM palmitate significantly increased mTOR and p70 S6K phosphorylation (403 ± 85.60 and 200 ± 42.55% of control, *p* < 0.0001, respectively) (Figure 6A–C). Treatment with RE alone did not affect the basal mTOR or p70 S6K phosphorylation (104 ± 13.71 and 82.12 ± 6.04% of control, respectively) while completely abolished the palmitate-induced phosphorylation of mTOR and p70 S6K (60 ± 20.53% and 90 ± 7.11% of control, *p* = 0.0002 and *p* = 0.0005, respectively), (Figure 6A–C). The total levels of mTOR and p70 S6K were not significantly changed by any treatment: P: 104 ± 3.01, 105 ± 5.83, RE: 93 ± 2.44, 97 ± 2.21 and RE + P: 88 ± 3.85, 92.22 ± 4.23% of control, respectively (Figure 6A–C).

### 3.6. Rosemary Extract increases the Phosphorylation of AMPK in the Presence of Palmitate

**Figure 7:**
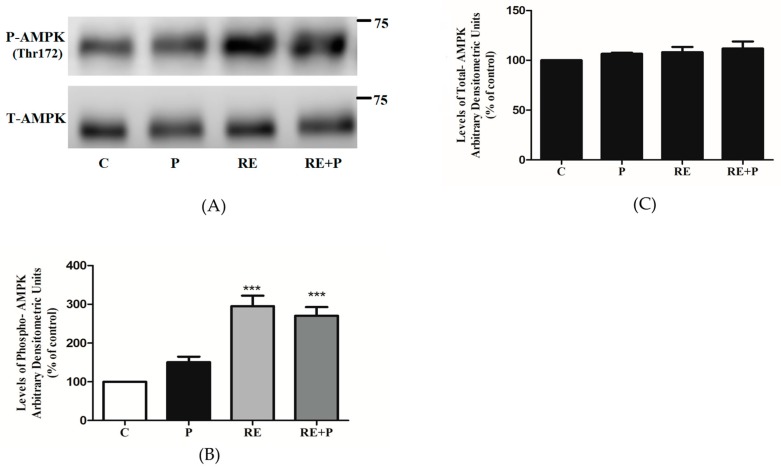
Effects of palmitate and RE on AMPK expression and phosphorylation. Fully differentiated myotubes were treated without (control, C) or with 0.2 mM palmitate (P) for 16 h in the absence or the presence of 5 μg/mL RE. After treatment, the cells were lysed, and SDS-PAGE was performed, followed by immunoblotting with specific antibodies that recognize phosphorylated Thr172 or total AMPK. Representative immunoblots are shown (**A**). The densitometry of the bands was measured and expressed in arbitrary units (**B**,**C**). The data are the mean ± SE of three separate experiments (*** *p* < 0.001 vs. control).

Previous studies by our group showed that rosemary extract and rosemary extract polyphenols increased glucose uptake and phosphorylated/activated AMPK in L6 muscle cells [38,48,49,50]. Here we investigated the chronic effect of RE on AMPK as well as the effect of RE on AMPK in an environment of elevated FFA. Treatment with 5 μg/mL RE significantly increased the phosphorylation of AMPK (295 ± 26.94% of control, *p* < 0.0001) (Figure 7A,B). Most importantly, RE increased phosphorylation of AMPK even in the presence of 0.2 mM of palmitate (270 ± 22.54% of control, *p* < 0.0001), (Figure 7A,B). Treatment with palmitate alone did not have any significant effect on the phosphorylation of AMPK (150 ± 14.32% of control). Furthermore, the total levels of AMPK were not affected by any treatment (P: 103 ± 8.63, RE: 99 ± 13.21, RE + P: 108 ± 9.33% of control) (Figure 7C).

## 4. Discussion

Obesity and elevated FFAs are highly correlated with insulin resistance and are major risk factors for the development of type 2 diabetes mellitus [19], a disease affecting millions of people globally. The search of compounds with the potential to counteract insulin resistance is the focus of many research groups worldwide and such compounds will provide huge benefits.

In the present study, we found that exposure of L6 myotubes to palmitate, to mimic the elevated plasma FFA levels seen in obesity in vivo, significantly decreased the insulin-stimulated glucose uptake indicating the induction of insulin resistance. These data are in agreement with previous studies showing that exposure of skeletal muscle cells to similar concentrations of palmitate induced insulin resistance [6,51,52,53]. Most importantly, in the presence of rosemary extract the palmitate-induced insulin resistance was prevented and the insulin-stimulated glucose uptake was restored to levels comparable to the response seen with insulin alone. These findings are the first to show that RE can counteract the palmitate-induced insulin resistance. It should be noted that although all the experiments in the present study were performed using the same batch of RE, in our lab we prepared a total of 3 different batches of RE using the same source of whole dried rosemary leaves (compliments of Sobey’s Mississauga, ON, Canada) and we tested them; all 3 batches gave us the same response, significantly increased L6 muscle cell glucose uptake and activated AMPK. We found that exposure of L6 cells to palmitate for 16 h increased Ser307 and Ser636/639 phosphorylation of IRS-1 in agreement with other studies showing increased Ser307 and Ser636/639 phosphorylation of IRS-1 by palmitate exposure in L6 [52,54] and C2C12 [55]. Our data are in agreement with in vivo animal studies showing increased serine phosphorylation of muscle tissue IRS-1 by high fat diet [15,17,56]. Increased phosphorylation of these serine residues of IRS-1 lead to a decreased PI3K-Akt downstream signaling and reduced glucose uptake [57]. Importantly our data show that treatment with RE prevented the palmitate-induced serine phosphorylation of IRS-1. This effect of RE is similar to metformin, the first line of treatment for T2DM, found to decrease the palmitate-induced Ser307 phosphorylation of IRS-1 in L6 muscle cells [58].

Furthermore, our data showed that exposure of the cells to palmitate significantly attenuated the insulin-stimulated phosphorylation of Akt. These data are in agreement with other in vitro studies using L6 [59], or C2C12 [60] cells and in vivo studies showing attenuation of the insulin-induced phosphorylation of Akt in isolated soleus muscle from animals fed a high-fat diet [61]. Interestingly, in the presence of RE the insulin-induced phosphorylation of Akt was restored indicating that RE has a potential to counteract the deleterious effects of palmitate and act similarly to metformin shown to counteract the effects of palmitate and restore insulin-induced Akt phosphorylation in L6 muscle cells [62].

Exposure of L6 muscle cells to palmitate significantly increased the phosphorylation of JNK in agreement with other studies in L6 [63] and C2C12 [64] muscle cells as well as findings from in vivo studies showing increased phosphorylation of JNK in muscle tissue from animals fed a high-fat diet [46,65]. Our data show that treatment with RE prevented the palmitate-induced phosphorylation of JNK in L6 muscle cells and are in agreement with a study showing quercetin, a polyphenol from the flavonoid group, to significantly attenuate the palmitate-induced phosphorylation of JNK in L6 muscle cells and in muscles obtained from ob/ob mice [63].

Furthermore, exposure of L6 cells to palmitate significantly increased the phosphorylation of mTOR and its downstream effector p70 S6K and treatment with RE abolished the palmitate effects. Although increased mTOR and p70 S6K phosphorylation by palmitate has been reported previously in L6 [66] and C2C12 cells [67] and in muscle tissue from animals fed a high-fat diet [66,68], our study is the first to show that RE has the potential to block these effects. Our data indicate the potential of RE, similar to metformin, to block the palmitate-induced mTOR and p70 S6K phosphorylation in C2C12 muscle cells [69].

Furthermore, we investigated the total and phosphorylated levels of AMPK. Previously, we found that treatment of L6 myotubes with RE [38] and the RE polyphenols carnosic acid (CA) [48], rosmarinic acid [49] and carnosol [50] significantly increased the phosphorylation of AMPK. In the present study, we found that 0.2 mM palmitate for 16 h did not affect AMPK phosphorylation or expression. Treatment with RE increased the phosphorylation/activation of AMPK even in the presence of palmitate, an effect similar to metformin which has been shown to phosphorylate/activate AMPK in the presence of palmitate in C2C12 and L6 muscle cells [62,69]. Studies have indicated that activation of AMPK significantly lowers the activity of mTOR and its downstream effector p70 S6K [70,71] and, therefore, the inhibition of mTOR and p70 S6K phosphorylation by RE treatment, seen in our study, may be mediated by AMPK. Studies using strategies to inhibit AMPK such as using an inhibitor of AMPK (Compound C) or siRNA techniques should be performed in the future to explore this further. To our surprise, exposure of the cells to RE and palmitate did not have a significant effect on the glucose uptake, indicating that in the presence of palmitate not only the acute insulin response was abolished but also the effect of RE is attenuated (Figure 2). It should be noted that RE in the presence of palmitate, resulted in a significant increase in AMPK phosphorylation and our data indicate that this increase was enough to abolish the palmitate-induced phosphorylation of mTOR and p70 S6K leading to a decrease in serine phosphorylation of IRS-1 but not sufficient to increase the glucose uptake in the cells (Figure 7, RE + P increased AMPK phosphorylation; Figure 2: RE + P no significant increase in glucose uptake). We have investigated previously the effects of RE, CA and RA on glucose transporters in GLUT4 and GLUT1 overexpressing cells and found no effect on glucose transporter translocation [38,48,49], and we had proposed that RE may increase glucose uptake by affecting GLUT3 translocation or by affecting glucose transporter activity. The lack of a significant increase in glucose uptake by RE in the presence of palmitate (Figure 2: RE + P) indicates that palmitate may affect a signaling step downstream of AMPK such as TBC1D1 that prevents the increase in glucose transporter activity/glucose uptake.

A limited number of studies have also examined the antidiabetic effects of RE and its polyphenols in vivo. In high-fat diet-induced diabetic mice, the administration of RE significantly decreased the fasting plasma glucose levels (72%), decreased total cholesterol (68%), total fat fecal excretion (1–2 fold) and body weight, thereby improving the lipid profile of the mice [39]. Another study found that RE enriched with CA significantly ameliorated high-fat diet-induced obesity and metabolic syndrome in mice [72]. The administration of RE enriched with CA in obese rats resulted in significant attenuation of TNFα and interleukin 1α indicating the anti-inflammatory effects of RE [73]. Additional studies showed that dietary supplementation of RE enriched with CA resulted in body weight and epidydimal fat reduction [74], as well as suppression of hepatic steatosis [75]. In high-fat diet-induced diabetic rats, the administration of RA dose-dependently ameliorated hyperglycemia and insulin resistance in addition to increasing GLUT4 translocation to the plasma membrane in muscle [36]. Moreover, a recent study conducted in humans administered dried rosemary leaf powder have shown significant improvement in the blood lipid profile, antioxidant levels, and decrease in fasting plasma glucose levels [76]. These studies demonstrate that RE and its polyphenols exhibit antihyperglycemic and antidiabetic properties in vivo and are in agreement with our findings. However, there are currently no studies that elucidate the mechanism involved in the effects of RE and its polyphenols. The present study found increased serine phosphorylation of IRS-1, and increased phosphorylation of mTOR, p70 S6K and JNK by palmitate and an effect of RE treatment to inhibit them and restore the insulin-stimulated Akt phosphorylation and the insulin-stimulated glucose uptake.

## 5. Conclusions

The prevalence of T2DM is constantly increasing and according to the International Diabetes Federation it is expected to affect 420 million people worldwide by the year 2040 [42]. Additionally, insulin resistance and T2DM are highly correlated with the development of other pathological states including cardiovascular disease and cancer [19]. As a result, new strategies to aid in the prevention and management of T2DM will provide huge benefits to our society. As previously indicated, increased levels of FFA and obesity mediate insulin resistance in muscle cells. The present study has shown that the exposure of muscle cells to the FFA palmitate, to mimic the elevated FFA levels seen in obesity, induced insulin resistance. Palmitate increased the serine phosphorylation of IRS-1 and phosphorylation of JNK, mTOR and p70 S6K, while the insulin-stimulated Akt phosphorylation and the insulin-stimulated glucose uptake were significantly reduced. Importantly, these effects of palmitate were attenuated by rosemary extract and the insulin-stimulated glucose uptake was restored. In addition, rosemary extract increased the phosphorylation/activation of the energy sensor AMPK, the activation of which has recently been recognized as a targeted approach to counteract insulin resistance and T2DM. Our study is the first to show that rosemary extract has the potential to counteract the palmitate-induced muscle cell insulin resistance, and further studies are required to explore its antidiabetic properties and to elucidate the exact cellular mechanisms involved.
